# Efficacy of Omalizumab in Indolent Systemic Mastocytosis

**DOI:** 10.1155/2019/3787586

**Published:** 2019-09-16

**Authors:** Calum Slapnicar, Martina Trinkaus, Lisa Hicks, Peter Vadas

**Affiliations:** ^1^Division of Allergy and Clinical Immunology, St. Michael's Hospital, University of Toronto, Toronto, Ontario, Canada; ^2^Division of Hematology, St. Michael's Hospital, University of Toronto, Toronto, Ontario, Canada

## Abstract

**Background:**

Systemic mastocytosis (SM) comprises a heterogeneous group of disorders characterized by the proliferation of clonal mast cells in skin and various internal organs. Omalizumab is an established, labelled therapy for allergic asthma and chronic urticaria, but its experience in the efficacy of SM is limited.

**Methods:**

A retrospective analysis of 6 patients diagnosed with indolent SM treated with omalizumab at St. Michael's Hospital between 2009 and 2018 is described. Reported frequency of anaphylaxis, baseline and follow-up tryptase levels, and SM-related symptoms were captured to measure the control of the disease.

**Results:**

Of the 5 patients who had experienced unprovoked anaphylaxis prior to treatment with omalizumab, 3 had no further episodes of anaphylaxis following initiation of omalizumab, while the remaining 2 patients had milder multisystem reactions. Significant improvement in cutaneous symptoms was also observed.

**Conclusion:**

These data suggest that omalizumab provides benefit to patients with SM who remain highly symptomatic in spite of treatment with conventional therapies.

## 1. Background

Systemic mastocytosis (SM) comprises a heterogeneous group of disorders characterized by accumulation of clonal mast cells (MCs) in various internal organs, including the bone marrow (BM), spleen, liver, and gastrointestinal tract (GI) as well as the skin [1–3]. In 2016, the World Health Organization revised the major and minor diagnostic criteria for SM [1]. Binding of the kit ligand, stem cell factor (SCF), to the kit receptor is important for the normal development and function of MCs from their hematopoietic progenitors [4]. Most individuals with SM carry a mutation at codon 816, wherein aspartic acid is replaced by valine (D816V), resulting in ligand-independent autophosphorylation of the kit receptor [4, 5]. Constitutive activation of MCs results in secretion of multiple MC-derived mediators, including histamine and prostaglandin D2. Alpha tryptase is released constitutively from MC and reflects the total body MC burden, whereas beta tryptase is a marker of MC activation [6, 7]. In Canadian laboratories, reports of tryptase levels reflects the total of both alpha and beta tryptase. Circulating IgE or cytokines as well as extrinsic factors such as physical stimuli, heat, alcohol, insect stings, or infection can all induce MC activation and, potentially, anaphylaxis [8, 9].

Classification of SM can fall into one of the five major subvariants: (i) indolent SM (ISM, the most common and carrying the most favourable prognosis); (ii) smoldering SM (SSM); (iii) SM with an associated nonmast cell clonal hematological disease (SM-AHN); (iv) aggressive SM (ASM); and (v) MC leukemia (MCL) [1, 10, 11]. SM is a rare disease with an annual incidence of 5–10 new cases per million population [12, 13].

SM has no cure apart from hematopoietic stem cell transplant [14], and treatment algorithms focus for the most part on symptom control. Treatment ladders are used in highly symptomatic patients to capture control [15]. These include use of H1 and H2 antagonists, leukotriene modifiers, and mast cell stabilizers, such as ketotifen and sodium cromoglycate [15]. In patients with a suboptimal response, omalizumab, a monoclonal anti-IgE antibody, may be considered [16, 17]. This therapy was licensed for use in patients with severe asthma and chronic spontaneous urticaria. However, a number of studies [16, 17] suggest that omalizumab may provide additional benefit for patients with SM with recurrent anaphylaxis despite maximal mast cell blockade.

Herein, we describe the efficacy of omalizumab in 6 patients with indolent SM, who experienced recurrent anaphylaxis refractory to maximal medical therapy.

## 2. Methods

Data were collected retrospectively on patients diagnosed with ISM and treated with omalizumab at our institution between January 2009 and May 2018. This study was approved by the St. Michael's Hospital Research Ethics Board. Only those patients who fulfilled the World Health Organization (WHO) diagnostic criteria for ISM were included. All patients underwent bone marrow biopsies for diagnosis. Those diagnosed with a different SM subvariant were excluded. Tryptase levels, frequency of anaphylaxis, and other mastocytosis-related symptoms were collated to assess control of symptoms. Severity of anaphylaxis was graded according to Brown [18].

## 3. Results

### 3.1. Patient 1

A 28-year-old male developed urticaria pigmentosa confirmed by skin biopsy in 2008. The number of skin lesions had been gradually increasing over the course of 6 years. A bone marrow biopsy confirmed the presence of ISM with c-kit D816V mutation and serum tryptase of 134 ng/ml. He complained of nausea, palpitations, and presyncope, which recurred every 2–4 weeks [19]. More severe multisystem reactions occurred approximately twice per year, usually triggered by respiratory tract infections, resulting in itchy skin, flushing, nausea, lightheadedness, severe joint pain, and mild throat constriction with no loss of consciousness. Symptoms were poorly controlled in spite of sodium cromoglycate, ketotifen, and cetirizine. In late 2014, treatment with omalizumab 150 mg at 4-week intervals was started (Table 1). In early 2015, due to a suboptimal response to therapy, his omalizumab dosage was increased to 300 mg every four weeks. Four months later, tryptase levels had fallen from 134 ng/ml to 84 ng/ml (Table 1), with complete control of all symptoms by seven months. A trial of dose reduction of omalizumab back to 150 mg every 4 weeks resulted in a return of episodic flushing, nausea, bone pain, palpitations, and diarrhea as well as 2 anaphylactic reactions, both requiring hospitalization within a span of 6 weeks. Basal tryptase levels had increased to 100 ng/ml. Omalizumab dosage was increased to 300 mg at 2-week intervals and later maintained at 4-weeks intervals. By early 2017, he had experienced only one reported flare of symptoms with facial flushing, dizziness, and diarrhea. In April 2018, the patient reported his first anaphylactic reaction in nearly two years, 3 months after omalizumab injections had been discontinued. The number of skin lesions of urticaria pigmentosa was not controlled by omalizumab, and later, he required ultraviolet therapy with a good clinical response.

### 3.2. Patient 2

A 49-year-old woman was diagnosed with c-kit-positive ISM with tryptase 11.4 ng/ml (Table 1). Symptoms of SM included daily headaches, sporadic flushing, itchy throat, mild coughing, loose stools, lightheadedness, and palpitations. No cutaneous manifestations of mastocytosis were observed. She was markedly intolerant to exercise, heat, and strong emotion, all of which triggered the symptoms in spite of medications, which included ranitidine, loratadine, cetirizine, ketotifen, and montelukast. In early 2017, she experienced 5 anaphylactic reactions, requiring emergency department visits and treatment with epinephrine as well as 5 milder reactions managed at home (Table 1). Omalizumab was started at 300 mg at 4-week intervals (Table 1). On omalizumab, multisystem reactions were entirely suppressed, with only breakthrough flushing once per week, loose stools roughly twice per month, and occasional lightheadedness. A follow-up one year later revealed complete resolution of symptoms with no adverse reactions to her medications and reduced tryptase to 8.3 ng/ml (Table 1).

### 3.3. Patient 3

A 51-year-old female with c-kit-positive ISM and tryptase 31.8 ng/ml (Table 1) reported intermittent episodes of shortness of breath, chest tightness, abdominal pain, and diarrhea. She had generalized lesions of urticaria pigmentosa involving her entire body, although numerous on her lower extremities. Ketotifen and ranitidine failed to control the symptoms. Omalizumab injections were started at 300 mg every two weeks (Table 1). At follow-up, her shortness of breath and chest tightness were controlled and GI symptoms had improved. Furthermore, her urticaria pigmentosa had improved and was now only observed on the lower abdomen and upper thighs. After the fourth cycle of omalizumab, she experienced an immediate multisystem reaction with respiratory distress and tongue swelling. A fifth cycle was attempted but again resulted in a similar reaction, leading to discontinuation of omalizumab therapy. At follow-up, she reported a worsening respiratory and functional status as well as 3-4 loose bowel movements per day. Basal tryptase levels had increased up to 41.2 ng/ml (Table 1).

### 3.4. Patient 4

A 40-year-old female with c-kit-positive ISM had daily flushing, nausea, abdominal discomfort, and loose stools 3-4 times per day. She had cutaneous lesions of telangiectasia macularis eruptiva perstans and tryptase 55.2 ng/ml (Table 1). She complained of debilitating bone pain, episodic presyncope, shortness of breath, and frequent palpitations. Ongoing medications included cetirizine, ranitidine, ketotifen, and cromolyn. Treatment with omalizumab 300 mg every 4 weeks was started (Table 1). After 3 injections, bone pain and GI symptoms had subsided. After 6 cycles of omalizumab, cutaneous lesions had decreased in number, daily palpitations and syncopal episodes had subsided, and tryptase had increased to 61.7 ng/ml (Table 1). She was subsequently able to discontinue both ketotifen and cromolyn with ongoing treatment with omalizumab.

### 3.5. Patient 5

A 32-year-old male with lesions of urticaria pigmentosa was diagnosed with c-kit-positive ISM. During the summer of 2015, while working outside, he experienced 3 Hymenoptera stings resulting in immediate onset of flushing and presyncope (Table 1). In spite of administration of epinephrine, he developed nausea and vomiting, generalized hives, and worsening presyncope. He was resuscitated in an emergency department but continued to experience daily diarrhea, numbness, and tingling sensation in his extremities, night sweats, severe headaches, and flushing upon strenuous physical activity. Because the symptoms were refractory to a combination of cetirizine, ranitidine, montelukast, and cromolyn, he was started on omalizumab 300 mg at 4-week intervals (Table 1). A follow-up 8 months later revealed improved symptomatic control and a fall in tryptase from 42.2 to 34 ng/ml (Table 1). However, he has continued to experience intermittent itching, flushing, and diarrhea. A follow-up in late 2018 revealed that he had chosen to discontinue omalizumab, with continued control of disease.

### 3.6. Patient 6

A 72-year-old female was diagnosed with c-kit-positive ISM. She had no cutaneous manifestations of mastocytosis. Her initial presentation was that of Hymenoptera anaphylaxis, with flushing, palpitations, and loss of consciousness (Table 1). She was treated with cetirizine and ranitidine but continued to experience occasional facial flushing, vomiting, lightheadedness, drenching night sweats, and bone pain. Given her history of venom anaphylaxis, omalizumab was started at 300 mg every 4 weeks (Table 1) for 14 months followed by rush venom desensitization [20, 21]. Symptoms of her underlying ISM resolved completely on ongoing omalizumab therapy. Tryptase had also fallen from 40.5 to 36.8 ng/ml.

## 4. Discussion

The current treatment of indolent systemic mastocytosis focuses on symptom control and suppression of mast cell mediator release [1]. Newer targeted agents such as avapritinib and midostaurin are only available for treatment of symptomatic ISM via clinical trials or through limited compassionate access programs. Neither drug was available for the patients described in this report. In accordance with other reports [22–26], treatment with omalizumab was effective in suppressing symptoms in patients with ISM, including recurrent anaphylaxis and other mast-cell-related symptoms refractory to maximal pharmacotherapy.

A number of studies [16, 17] describe omalizumab as being the most effective in controlling frequency and severity of recurrent anaphylaxis in ISM. In one cohort, ten of twelve patients treated with omalizumab 300 mg at 4-week intervals had a good therapeutic response [16], whereas two of twelve patients were maintained on 150 mg at 2- and 4-week intervals. In keeping with these reports, our patients had optimal control of disease manifestations on a maintenance dose of 300 mg at 4-week intervals. One patient achieved complete control on this regimen and was subsequently tapered down to 150 mg every 4 weeks, leading to the return of unprovoked anaphylaxis, and daily symptoms attributed to uncontrolled disease.

Not only did patients experience a reduction in frequency and severity of anaphylaxis but also four of six patients had a decline in tryptase levels (134 ng/ml to 84.1 ng/ml; 11.4 ng/ml to 8.3 ng/ml; 42.2 ng/ml to 34 ng/ml; 40.5 ng/ml to 36.8 ng/ml). One patient had an increase (55.2 ng/ml to 61.7 ng/ml), and the patient who discontinued omalizumab due to an adverse reaction had an increase from baseline (31.8 ng/ml to 41.2 ng/ml). Chang et al. [27] present evidence that IgE binding to FcεRI, but without cross linking, will modulate the mast cell function. Specific effects include increased mast cell proliferation and survival and a reduction in the threshold of degranulation of mast cells with a corresponding increase in sensitivity to various allergens. Conversely, depletion of IgE by omalizumab will result in reduction of the ability of mast cells to degranulate, with a concomitant fall in mediator release.

Although the results of this study are encouraging, this study is a retrospective, single-center study with a small cohort of patients. Nonetheless, the results in this small group of patients with symptoms refractory to routine pharmacotherapy are promising and suggest that omalizumab should be considered in highly symptomatic patients with ISM. Future prospective studies of omalizumab or other potential therapies would benefit by use of methodologies such as ISM-specific quality-of-life questionnaire developed by van Anrooij et al. [28].

This case series describes the response to therapy in 6 patients with ISM treated with omalizumab. These data lend further support to the use of omalizumab in treatment of patients with clonal mast cell disorders, including mastocytosis and monoclonal mast cell activation syndrome [29], adding to the existing evidence in the literature, supporting the efficacy of omalizumab in highly symptomatic patients.

## Figures and Tables

**Table 1:** Clinical characteristics and response to therapy of patients diagnosed with indolent systemic mastocytosis.

Patient #	Age	Gender	Disease duration	Optimal omalizumab dosage	Tryptase preomalizumab therapy	Tryptase during omalizumab therapy	Annualized frequency of anaphylaxis before treatment with omalizumab	Annualized frequency of anaphylaxis on optimal dose of omalizumab
1	38	M	10 years	150 mg·q. 4 weeks	134 ng/ml	84.1 ng/ml	2	2
2	51	F	3 years	300 mg·q. 4 weeks	11.4 ng/ml	8.3 ng/ml	60	0
3^1^	55	F	4 years	300 mg·q. 2 weeks	31.8 ng/ml	N/A	—	—
4	42	F	2 years	300 mg·q. 4 weeks	55.2 ng/ml	61.7 ng/ml	1	0
5	42	M	10 years	300 mg·q. 4 weeks	42.2 ng/ml	34 ng/ml	1	0
6	75	F	3 years	300 mg·q. 4 weeks	40.5 ng/ml	36.8 ng/ml	1	0

^1^This patient had poorly controlled manifestations of ISM but no history of anaphylaxis.
